# Symmetry After Breast Reconstruction Surgery: A Comparison of Immediate vs. Delayed-Immediate Breast Reconstruction Using Smartphone-Based 3D Surface Imaging

**DOI:** 10.3390/jcm14217622

**Published:** 2025-10-27

**Authors:** Robin Hartmann, Nikolas Chrobot, Christian Festbaum, Michael Alfertshofer, Katharina Theresa Obermeier, Wenko Smolka, Tobias Ettl, Lukas Prantl, Vanessa Brébant

**Affiliations:** 1 Department of Oral and Maxillofacial Surgery and Facial Plastic Surgery, Ludwig-Maximilians-University (LMU), Lindwurmstrasse 2a, 80337 Munich, Germany; 2 University Center of Plastic, Aesthetic, Hand and Reconstructive Surgery, University Hospital Regensburg, Franz-Josef-Strauß-Allee 11, 93053 Regensburg, Germanylukas.prantl@klinik.uni-regensburg.de (L.P.); 3 Department of Plastic and Hand Surgery, Technical University Munich, 81675 Munich, Germany; 4 Department of Oral and Maxillofacial Surgery, University Hospital Regensburg, Franz-Josef-Strauß-Allee 11, 93053 Regensburg, Germany

**Keywords:** three-dimensional surface imaging, smartphone-based surface imaging, breast reconstruction surgery, stereophotogrammetry, plastic surgery

## Abstract

**Background:** Breast reconstruction surgery (BRS) is a vital coping mechanism for patients undergoing mastectomy. Various methods have been introduced, including immediate and delayed-immediate BRS. This study employs a smartphone-based approach for three-dimensional (3D) surface imaging to compare outcomes after immediate vs. delayed-immediate BRS. **Methods:** Twenty-six patients who underwent BRS using the deep inferior epigastric perforator (DIEP) flap at our institution from 1 October 2018 to 1 October 2023 were included in this study. Thirteen patients underwent immediate BRS and thirteen underwent delayed-immediate BRS. Following successful BRS, each patient underwent a digital anthropometric examination that included 14 measurements and the calculation of the symmetry index (SI) using the iPhone 15 Pro along with the 3D Scanner App and the Vectra Analysis Module (VAM). Measurements were subsequently compared between immediate and delayed-immediate BRS using the *t*-test for independent samples. **Results:** For 11 of 14 measurements, no significant differences were detected between immediate and delayed-immediate BRS. The SI did not differ significantly between immediate (M = 0.85) and delayed-immediate (M = 0.88) BRS (*t*-test for independent samples; *p* = 0.23, n = 26, two-tailed). Additionally, no significant differences were found between patients’ age, height, weight, BMI, time since first diagnosis, and flap weight using a *t*-test for independent samples. **Conclusions:** No statistically significant differences in breast symmetry were detected between immediate and delayed-immediate reconstruction in this cohort. This study supports the integration of smartphone-based 3D imaging into routine plastic surgery.

## 1. Introduction

Female breast cancer represents the second most common cancer worldwide, accounting for 11.6% of all global cancer cases [1]. Despite rising incidence, recent statistics report declines in breast cancer mortality from 2% to 3% annually during the 1990s and 2000s to 1% annually from 2013 to 2021 [2]. Mastectomy is necessary for approximately one-third of patients undergoing cancer treatment [3]. With increasing 5-year survival rates (74.8% in 1975 vs. 91.3% in 2015), procedures such as breast reconstruction surgery (BRS) have gained increasing importance, as they provide an important coping tool for patients undergoing mastectomy [4]. Several BRS techniques have been established as standard clinical practice in plastic surgery, including the immediate and delayed-immediate approaches [3,5,6,7,8].

Immediate reconstruction is performed at the time of mastectomy [6]. In contrast, the delayed-immediate approach involves a mastectomy with a subsequent insertion of a tissue expander, followed by definitive reconstruction if no radiation is needed or expander deflation for patients requiring radiation; the final reconstruction occurs post therapy [5]. This approach is primarily used for patients whose need for postmastectomy radiation therapy (PMRT) is preoperatively uncertain [9]. Some studies have evaluated the aesthetic outcomes of patients undergoing delayed-immediate BRS [9,10]. However, there is limited data comparing postoperative optical symmetry between immediate vs. delayed-immediate BRS.

For both surgeons and patients, obtaining symmetry is a major goal in BRS, irrespective of the technique employed [11]. Consequently, several studies have utilized three-dimensional (3D) surface imaging to assess optical symmetry after BRS [12,13,14,15]. Novel state-of-the-art approaches, including smartphone-based 3D surface imaging for outcome evaluation in breast surgery, have emerged due to their low cost, making them accessible to a broader range of surgeons worldwide [16].

This study aims to compare the optical symmetry of outcomes after immediate and delayed-immediate BRS using the deep inferior epigastric perforator (DIEP) flap through smartphone-based 3D surface imaging.

## 2. Material and Methods

### 2.1. Study Protocol

This trial was designed as a single-center retrospective–prospective cohort study conducted at the Department of Plastic Surgery at St. Josef Hospital in Regensburg, Germany. Prior to the commencement of the study, ethical approval was obtained from the local ethics committee (20-1653-3-101). A total of 26 participants who underwent BRS between 1 October 2018 and 1 October 2023 were evaluated. Patients who experienced flap loss, exhibited sensitivity to plaster, or declined to participate in the anthropometric assessment were excluded from the study.

### 2.2. Patient Preparation

Patients were invited to participate in a digital anthropometric examination during their regular follow-up visits. In accordance with a previously described protocol, participants were positioned in a standardized upright posture at a scanning distance of approximately 30–40 cm under uniform ambient lighting. In case of motion artifacts or failed scans, the acquisition was immediately repeated. Subsequently, 14 key anatomical landmarks were marked using colored stickers in accordance with a previously described methodology [12,17]. Additionally, four landmarks on the upper arms, throat, and upper abdomen were identified for subsequent processing and cropping. If a patient did not receive nipple–areola complex (NAC) reconstruction, a healthcare professional approximated the prospective position of the NAC using an additional landmark according to a standard protocol [12].

**Figure 1:**
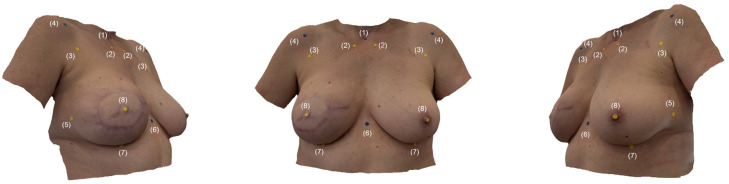
*Landmarks*: Appearance of a 50-year-old patient after immediate breast reconstruction (R); the iPhone 15 Pro (Apple Inc., Cupertino, CA, USA) using the “3D-Scanner App” V2.1.2 (Laan Consulting Corp., New York, NY, USA) was utilized to create the SM. (1) Sternal Notch (SN), (2) Medial Upper Breast Pole (MUBP) (R+L), (3) Upper Lateral Breast Pole (LUBP) (R+L), (4) Coracoid Process (CP) (R+L), (5) Lateral Breast Pole (LaBP) (R+L), (6) Xiphoid (Xi), (7) Lower Breast Pole (LBP) (R+L), and (8) Nipple (N) (R+L).

Figure 1 provides an overview of all landmarks.

### 2.3. Three-Dimensional Data Acquisition

Three-dimensional surface models (SMs) were obtained using the iPhone 15 Pro and the 3D Scanner App V2.1.2 (Laan Consulting Corp., New York, NY, USA). The app’s photo mode was utilized to capture the patients’ surface information. This setup utilizes Apple’s light detection and ranging (LiDAR) sensor combined with photogrammetry. LiDAR uses time-of-flight measurements to determine the distance (or depth) between an object and the sensor [18]. Previous studies indicate that LiDAR-based anthropometric measurements exhibit a relative technical error of measurement (rTEM) ranging from 2.99% to 5.19% compared to reference data [19].

**Figure 2:**
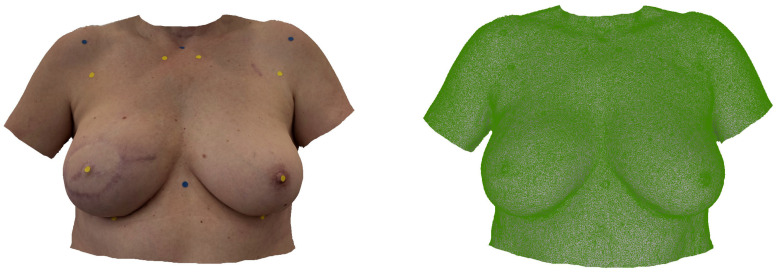
*Smartphone-based SM*: Appearance of a 50-year-old patient after immediate breast reconstruction (R) using a DIEP flap weighing 850 g. The iPhone 15 Pro was employed along with the 3D Scanner App (Laan Consulting Corp., USA) to create the SM. The left image displays the SM with applied texture, while the right image shows the SM without texture.

**Figure 3:**
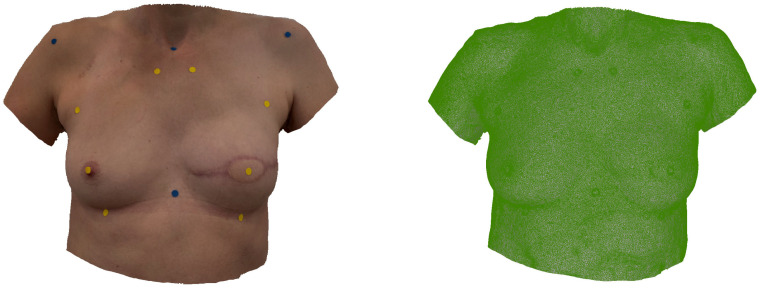
*Smartphone-based SM:* Appearance of a 47-year-old patient after delayed-immediate breast reconstruction (R) using a DIEP flap weighing 415 g. The iPhone 15 Pro was utilized alongside the 3D Scanner App (Laan Consulting Corp., New York, NY, USA) to create the SM. The left image presents the SM with applied texture, and the right without texture.

Figure 2 shows a smartphone-based SM used for anthropometric assessment in a 50-year-old patient after immediate BRS. Figure 3 shows a smartphone-based SM of a 47-year-old patient after delayed-immediate BRS.

### 2.4. Digital Anthropometric Examination

Smartphone-based SMs were exported as Wavefront OBJ files. Using CloudCompare (http://cloudcompare.org/), the SMs were cropped at the upper arms, throat, and upper abdomen. They were then imported into the Vectra Analysis Module (VAM) for further analysis.

According to a previously described protocol, 14 anthropometric measurements were performed by a healthcare professional: (1) Sternal Notch (SN)—Nipple (N) (R), (2) Lower Breast Pole (LBP)—Nipple (N) (R), (3) Upper Breast Pole (UBP)—Nipple (N) (R), (4) Xiphoid (Xi)—Nipple (N) (R), (5) Lateral Breast Pole (LaBP)—Nipple (N) (R), (6) Sternal Notch (SN)—Nipple (N) (L), (7) Lower Breast Pole (LBP)—Nipple (N) (L), (8) Upper Breast Pole (UBP)—Nipple (N) (L), (9) Xiphoid (Xi)—Nipple (N) (L), (10) Lateral Breast Pole (LaBP)—Nipple (N) (L), (11) Breast width (R), (12) Inframammary Fold (IMF)-Length (R), (13) Breast width (L), and (14) IMF-Length (L). Subsequently, the data were subjected to analysis using the symmetry index (SI) (15). The SI synthesizes 14 anthropometric measurements into a single value representing optical symmetry, as previously described [12,17]. The analysis entails a comparison of the minimum and maximum anthropometric values of the left and right breasts. The SI is scored on a scale from 0 to 1, with 0 indicating the poorest symmetry and 1 indicating the best symmetry. The SI can also be expressed as a percentage by multiplying the score by 100.

**Figure 4:**
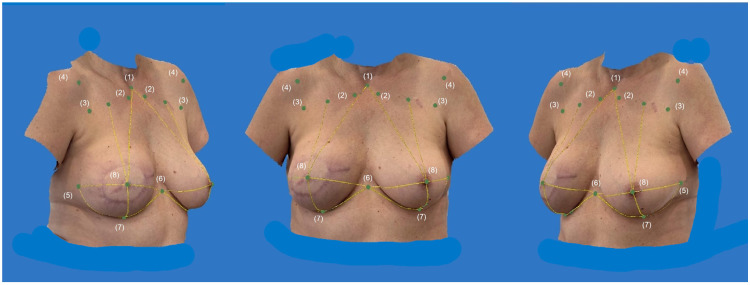
*Digital anthropometric examination*: Presentation of the anthropometric assessment on a 3D SM of a 50-year-old patient after immediate breast reconstruction (right) using a DIEP flap weighing 850 g. Measurements (1)–(14) were conducted using the VAM, and the SI was calculated with Microsoft Excel. Landmarks (1)–(8) are highlighted bilaterally on the SM to illustrate the key reference points used for the analysis.

Figure 4 presents the anthropometric assessment on a 3D SM of a 50-year-old patient after immediate breast reconstruction (R) using a DIEP flap weighing 850 g. The measurements (1)–(14) were conducted using the Vectra Analysis Module, and the symmetry index (SI) was calculated with Microsoft Excel.

### 2.5. Statistical Analysis

IBM SPSS Statistics 29 (SPSS Inc., Chicago, IL, USA) was utilized for statistical analysis. Data normality was assessed using the Kolmogorov–Smirnov test. Following confirmation of normality, independent samples *t*-tests were conducted to compare the means of measurements (1)–(14) and the SI between groups. Additionally, comparisons of age, height, weight, BMI, time since first diagnosis, and flap weight were performed using independent samples *t*-tests to evaluate significant differences among these variables. Homogeneity of variances was assessed with Levene’s test. Cohen’s d was calculated as a standardized effect size for each comparison. To assess potential confounding effects of bilateral reconstruction and contralateral breast reduction, a sensitivity analysis was performed excluding five affected patients. The SI was reanalyzed descriptively across groups. It had been defined a priori as the primary endpoint. The 14 individual anthropometric measurements were treated as secondary, exploratory endpoints to assess potential localized shape differences between groups. Due to the exploratory character of the study and the limited sample size, no formal multiplicity corrections were applied across all secondary endpoints.

## 3. Results

### 3.1. Patient Demographics

The cohort included 26 female participants, all of whom underwent BRS with a DIEP-flap. Thirteen patients underwent delayed-immediate BRS, while thirteen received immediate BRS. The mean age in the immediate group was M = 48.3 years (SD = ±6.6), mean height M = 166 cm (SD = ±6 cm), mean weight M = 78 kg (SD = ±15.5 kg), and mean BMI M = 28.2 (SD = ±4.6). The mean time between surface imaging and first diagnosis was 5.3 years (SD = ±3.6). The mean flap weight was 729 g (SD = ±379 g); Table 1. Eleven patients underwent unilateral BRS, while two underwent bilateral BRS. In six patients, BRS was performed on the right side, in five BRS was performed on the left side, while two patients underwent BRS on both sides. Three patients underwent skin-sparing mastectomy and ten patients underwent modified radical mastectomy. Nine patients underwent NAC reconstruction. Ten patients did not undergo contralateral breast reduction, while three underwent contralateral breast reduction surgery. Nine patients underwent PMRT, while four patients did not undergo PMRT.

The mean age in the delayed-immediate group was M = 53.1 years (SD = ±9.4), mean height M = 169 cm (SD = ±6 cm), mean weight M = 72 kg (SD = ±13.9 kg), and mean BMI M = 25.4 (SD = ±4.5). The mean time between surface imaging and first diagnosis was 5.7 years (SD = ±4.1). The mean flap weight was 516 g (SD = ±159 g) (Table 1). All 13 patients underwent unilateral BRS. In seven patients, BRS was performed on the right side, while six patients received BRS on the left side. Two patients underwent skin-sparing mastectomy, one patient underwent nipple-sparing, and ten patients underwent modified radical mastectomy. Nine patients underwent NAC reconstruction. Ten patients underwent PMRT, while three patients did not undergo PMRT. No patient underwent contralateral breast reduction surgery. No significant differences in age, height, weight, BMI, time since first diagnosis, or flap weight were detected using *t*-tests for independent samples (Table 2).

### 3.2. Anthropometric Examination and Symmetry Index

Table 3 presents the outcomes of the anthropometric examination. For 11 out of 14 measurements, no significant differences were detected between immediate and delayed-immediate BRS. A significant difference between the immediate group (M = 14.3 cm) and delayed-immediate group (M = 10.9 cm) was detected for measurement (5) LaBP—N (R) (*t*-test for independent samples; *p* < 0.001, two-tailed) (Table 4). In addition, values for measurement (11), Breast width (R), differed significantly between immediate (M = 27.1 cm) and delayed-immediate (M = 23.2 cm) BRS (*t*-test for independent samples; *p* = 0.01, two-tailed) (Table 4). Additionally, values for measurement (12), IMF-Length (R), differed significantly between immediate (M = 25.7 cm) and delayed-immediate (M = 22.7 cm) BRS (*t*-test for independent samples; *p* = 0.03, two-tailed) (Table 4).

However, values for the SI did not differ significantly between immediate (M = 0.85) and delayed-immediate (M = 0.88) BRS (*t*-test for independent samples; *p* = 0.23, two-tailed) (Table 4). Exclusion of bilateral and contralateral reduction cases (n = 5) did not alter the direction or interpretation of the group-level differences in the SI.

## 4. Discussion

The present trial compares two standard approaches for BRS utilizing smartphone-based surface imaging. Although no statistically significant differences in general symmetry (SI) were found, three anthropometric measurements displayed statistically significant differences. These measurements included two curved measurements (IMF-Length (R) and Breast width (R)), as well as the LaBP—N (R). The differences in these three measurements may be attributed to slight variations in laterality between the two groups. While patients exhibited comparable body habitus, as evidenced by the lack of significant differences in age, height, weight, BMI, time since first diagnosis, and flap weight, anthropometric measurements may still vary between the groups. Scar placement and scar quality were not investigated in the present study. However, they can significantly influence postoperative symmetry. Both reconstruction techniques aim for inconspicuous scarring, in contrast to secondary reconstruction, which can result in more extensive and often less predictable scarring. Therefore, scar placement was not evaluated as part of this analysis, since minimizing visible scarring represents a fundamental aim of both reconstruction techniques.

Moreover, while two patients in the immediate group underwent bilateral BRS, all patients in the delayed-immediate group underwent unilateral BRS. Three patients in the immediate group underwent contralateral breast reduction, whereas no patients in the delayed-immediate group had this procedure. These factors may also account for the observed differences in measurements (5), (11), and (12). Although bilateral and contralateral reduction cases occurred only in the immediate group, the findings remained consistent after exclusion, supporting the robustness of the main result.

Notably, three anthropometric measures on the right side (LaBP—N, Breast width, and IMF-Length) showed statistically significant differences between groups, despite a non-significant SI. These localized discrepancies may reflect clinical and procedural imbalances, such as the higher proportion of unilateral reconstructions and contralateral reductions in the immediate group. In addition, PMRT may induce laterality-specific fibrosis or scar behavior, particularly affecting the right side, which was more commonly affected in our cohort. These findings must be acknowledged when interpreting this study’s results.

In addition, the general limitations of smartphone-based surface imaging must be acknowledged. Previous studies have reported challenges in accurately assessing anatomical features in concave spaces, such as the orbital region [20]. The IMF is a concave space in most SMs. In their comparative anthropometric analysis of SMs obtained from the iPhone X and the Vectra H2, Rudy et al. noted the greatest landmark-to-landmark distance deviation for the IMF width. These findings align with the present study’s observations regarding discrepancies in IMF-Length and may help explain the observed differences [16]. Despite these limitations, both groups were stratified by age, height, weight, BMI, time since first diagnosis, and flap weight. Both groups exhibited a similar ratio of patients receiving PMRT, with ten patients receiving it in the delayed-immediate group and nine patients in the immediate group. Against this background, both methods for BRS demonstrated no statistically significant difference in general symmetry (SI).

Other drawbacks involve the SI. While it integrates 14 anthropometric values into a single dimensionless score, its clinical interpretability remains unclear. To ensure clinical relevance, future studies should aim to refine the index by validating it against perceptual assessments of symmetry. Given that the SI is not yet validated as a clinically perceptible measure, the study was not powered to detect small differences in symmetry. Accordingly, the results should be interpreted as indicative of directional tendencies rather than definitive quantitative thresholds. Furthermore, the inter-rater and intra-rater reliability of the SI were not evaluated, which should be addressed in future investigations. Additionally, the modest sample size limits the statistical power of this study. Cohen’s d is reported for all comparisons to convey effect magnitude. Future studies with larger cohorts may confirm this study’s findings.

This study employed an innovative, state-of-the-art technique for BRS that has the potential to become accessible to a broader range of surgeons globally due to its cost-effectiveness. Currently, few sophisticated systems, such as Crisalix (Crisalix SA, Lausanne, Switzerland), operate on smartphone/tablet-based applications [21]. When utilizing smartphone-based applications for anthropometric assessments, it is essential to critically assess the method’s accuracy. The accuracy of smartphone-based surface imaging remains a topic of ongoing research and has been evaluated differently by various authors [16,20,21,22,23,24,25,26,27]. The majority of authors, however, report sufficient accuracy for clinical use [22,24,28]. In their meta-analysis, Quinzi et al. reported an accuracy range for smartphone-based surface imaging of 0.460 to 1.400 mm, while stationary stereophotogrammetry systems ranged from 0.087 to 0.860 mm and portable stereophotogrammetry scanners from 0.150 to 0.849 mm [28]. The authors concluded that the method showed sufficiently reliable accuracy for clinical application [28]. Rudy et al. confirmed these findings for smartphone-based breast assessment. In their comparative study, the authors analyzed 20 breasts captured using both the iPhone X and the Vectra H2 system, employing colormap analysis and measuring distances across models between key anatomical landmarks. When assessing absolute differences between the two imaging methods, the average discrepancies in distances between key landmarks were found to be under 1 mm. The colormap analysis revealed an average error of 1.53 mm, with a mean of 0.53 mm and an SD of ±1.81 mm. Bland–Altman analysis indicated a mean difference of 0.13 mm, with an agreement interval ranging from −1.90 to 2.17 mm [16]. While the 3D scanning protocol followed a standardized clinical workflow, duplicate scans were not performed for formal repeatability assessment. This limitation should be addressed in future studies to further validate the robustness of the workflow in real-world settings. Another potential limitation concerns the approximation of the NAC in unreconstructed cases. Although this may introduce minor bias, the estimation followed established anatomical references and represents an acceptable clinical approximation.

Additionally, accuracy may vary significantly among different smartphone applications, and touchscreen-based anthropometry may not yet match the precision of established systems [23,29]. Consequently, this study exported SMs to an established system for computer-based measurements. This method may reduce measurement error. However, there is currently a lack of smartphone-based applications that may be utilized for automated anthropometry of the human body. Future studies should introduce producer-independent software for smartphone-based automated anthropometry that can be used for outcome assessment in BRS.

Both reconstruction methods achieved comparably high symmetry in the 3D analysis, indicating that the choice of timing may primarily depend on oncologic and patient-specific factors rather than aesthetic concerns. Moreover, smartphone-based 3D surface imaging proved easily integrable into clinical workflows, offering a cost-efficient and objective tool for outcome assessment. The localized right–left differences may reflect case-related factors, such as unilateral reconstructions, contralateral reductions, or PMRT-associated scar behavior, rather than methodological bias. Reliable data acquisition requires a standardized setup, including an upright position, a standardized scanning distance, uniform lighting, and repetition of scans in case of motion artifacts. This should be considered when implementing smartphone-based 3D surface imaging in clinical practice.

Both methods yielded comparable anthropometric outcomes using this novel approach. The integration of smartphone-based 3D surface imaging for outcome assessment has contributed to the broader implementation of this technology in routine clinical practice within plastic surgery, ultimately enhancing patient care in BRS. By enabling more surgeons globally to adopt this technique, the availability and accessibility of advanced imaging solutions may be improved.

## 5. Conclusions

This study comprehensively examines two well-established techniques for BRS using smartphone-based anthropometry. Using a novel smartphone-based approach, no significant difference in outcome symmetry was found between immediate and delayed-immediate reconstruction. By applying this technique, this study aims to contribute to the application of smartphone-based 3D surface imaging in clinical practice within plastic surgery and thus advance its further development. As technological advancements continue, plastic surgeons remain at the forefront of adopting innovative, cutting-edge methods to optimize patient outcomes.

## Figures and Tables

**Table 1:** Demographic data: Demographic characteristics of immediate and delayed-immediate groups—comparative analysis of age (years), height (cm), weight (kg), BMI (kg/m^2^), time since first diagnosis (years), and flap weight (g).

	Demographic Data							
*Variables*	Immediate (n = 13)				Delayed-Immediate (n = 13)			
	Min.	Max.	Mean	SD	Min.	Max.	Mean	SD
Age	32.0	58.0	48.3	6.6	38.0	64.0	53.1	9.4
Height	160	172	166	6	158	180	169	6
Weight	60.0	116.0	78	15.5	58.0	110.0	72	13.9
BMI	24.1	39.7	28.2	4.6	20.3	37.6	25.4	4.5
Time since first diagnosis	2.5	16.3	5.3	3.6	1.9	16.0	5.7	4.1
Flap weight	453	1550	729	379	250	857	516	159

**Table 2:** T-test for independent samples: Comparison of immediate and delayed-immediate groups. Comparative analysis of age (years), height (cm), weight (kg), BMI (kg/m^2^), time since first diagnosis (years), and flap weight (g). Data analysis was performed using IBM SPSS 29.

		T-Test for Independent Samples and Mean Values							
*Variables*									
	Mean		Levene’s Test			95% CI		T-test	Cohen’s d
	Immediate	Delayed-Immediate	*p*	Mean Diff.	Std. Error Diff.	Lower	Upper	*p*	Point Estimates
Age	48.3	53.1	0.09	−4.8	3.2	−11.3	1.8	0.15	0.81
Height	166	169	0.52	−3.0	2.3	−7.5	1.75	0.21	−0.5
Weight	78	72	0.58	5.6	5.8	−6.4	17.5	0.35	0.38
BMI	28.2	25.4	0.81	2.9	1.8	−0.8	6.6	0.12	0.63
Time since first diagnosis	5.3	5.7	0.49	−0.4	1.5	−3.6	2.8	0.80	−0.51
Flap weight	729	516	0.16	212.7	127.6	−57.9	483.3	0.12	−0.10

**Table 3:** Descriptive statistics: Anthropometric measurements, including minimum, maximum, mean, SD, and SI, are provided for smartphone-based measurements (1)–(14). Anthropometric measurements are in centimeters (cm). Data analysis was performed using IBM SPSS 29.

	Descriptive Statistics							
*Variables*	Immediate (n = 13)				Delayed-Immediate (n = 13)			
	Min.	Max.	Mean	SD	Min.	Max.	Mean	SD
(1) SN—N (R)	19.7	36.9	24.8	4.3	17.4	27.3	23.0	3.0
(2) LBP—N (R)	6.8	12.8	9.0	1.8	5.6	9.8	7.9	1.3
(3) UBP—N (R)	12.8	29.3	17.9	4.2	10.0	20.4	16.3	3.2
(4) Xi—N (R)	9.9	17.4	12.8	2.1	9.0	14.6	12.3	1.5
(5) LaBP—N (R)	8.9	19.4	14.3	2.5	8.8	14.3	10.9	1.7
(6) SN—N (L)	18.7	29.4	24.6	3.1	18.4	29.6	24.3	3.8
(7) LBP—N (L)	5.8	11.9	8.4	1.7	6.1	10.2	8.0	1.3
(8) UBP—N (L)	11.4	22.3	17.4	3.3	11.1	23.6	17.8	3.9
(9) Xi—N (L)	10.3	14.9	13.4	1.2	9.3	16.3	13.2	2.0
(10) LaBP—N (L)	8.7	16.2	13.1	2.5	9.3	14.9	11.8	1.9
(11) Breast width (R)	20.2	36.9	27.1	3.8	17.9	27.4	23.2	2.7
(12) IMF-Length (R)	20.7	37.5	25.7	4.3	18.7	25.6	22.7	2.3
(13) Breast width (L)	19.0	29.8	26.4	3.1	20.3	30.5	25.1	3.4
(14) IMF-Length (L)	16.8	30.9	24.9	3.6	20.0	29.4	24.2	3.2
(15) SI	0.74	0.97	0.85	0.06	0.78	0.97	0.88	0.06

**Table 4:** T-test for independent samples: Comparison of immediate and delayed-immediate groups. Means in centimeters (cm), smartphone-based measurements (1)–(14), and the SI (15); data analysis was performed using IBM SPSS 29.

T-Test for Independent Samples and Mean Values									
*Variables*									
	Mean		Levene’s Test			95% CI		T-Test	Cohen’s d
	Immediate	Delayed-Immediate	*p*	Mean Diff.	Std. Error Diff.	Lower	Upper	*p*	Point Estimates
(1) SN—N (R)	24.8	23.0	0.71	1.7	1.5	−1.3	4.7	0.24	0.5
(2) LBP—N (R)	9.0	7.9	0.52	1.2	0.6	−0.1	2.5	0.07	0.5
(3) UBP—N (R)	17.9	16.3	0.71	1.6	1.4	−1.4	4.6	0.27	0.6
(4) Xi—N (R)	12.8	12.3	0.13	0.5	0.7	−1	2.1	0.46	0.7
(5) LaBP—N (R)	14.3	10.9	0.46	3.4	0.8	1.6	5.1	<0.001	0.7
(6) SN—N (L)	24.6	24.3	0.30	0.3	1.3	−2.5	3.1	0.83	0.9
(7) LBP—N (L)	8.4	8.0	0.32	0.4	0.6	−0.8	1.7	0.47	0.4
(8) UBP—N (L)	17.4	17.8	0.36	−0.4	1.4	−3.3	2.5	0.77	0.4
(9) Xi—N (L)	13.4	13.2	0.06	0.1	0.6	−1.2	1.5	0.82	0.5
(10) LaBP—N (L)	13.1	11.8	0.43	1.2	0.9	−0.5	3	0.17	0.3
(11) Breast width (R)	27.1	23.2	0.67	3.9	1.3	1.2	6.6	0.01	0.3
(12) IMF-Length (R)	25.7	22.7	0.23	3.1	1.4	0.2	5.9	0.03	0.4
(13) Breast width (L)	26.4	25.1	0.44	1.3	1.3	−1.3	3.9	0.31	1.6
(14) IMF-Length (L)	24.9	24.2	0.62	0.7	1.3	−2.1	3.4	0.61	1.5
(15) SI	0.85	0.88	0.36	−0.03	0.02	−0.08	0.02	0.23	2.0

## Data Availability

The data analyzed in this study includes photographs of individuals and is therefore not publicly available. Informed consent for the use of individual photographs was obtained from every participant depicted in this manuscript.
